# Hepatic Angiomyolipoma in Patients With Inflammatory Breast Cancer: A Case Report

**DOI:** 10.7759/cureus.62651

**Published:** 2024-06-18

**Authors:** Abdulaziz Alotaibi, Samer AlGhazawi, Meshari Alghthami, Rofal Alqurashi, Ibrahim Alibrahim, Amjad Althagafi, Abdullah Alzeiyadi

**Affiliations:** 1 Hepatobiliary Surgery, Al Hada Armed Forces Hospital, Taif, SAU; 2 General Surgery, Al Hada Armed Forces Hospital, Taif, SAU; 3 College of Medicine, Umm Al-Qura University, Makkah, SAU

**Keywords:** general surgery, rare disease, inflammatory breast cancer, hepatology, hepatic angiomyolipoma

## Abstract

Hepatic angiomyolipoma (HAML) is a rare tumor comprising adipose tissue, smooth muscle cells, and blood vessels. On the other hand, inflammatory breast cancer (IBC) is a rare and severe form of breast cancer that progresses quickly and presents as breast inflammation. It is incredibly unusual for HAML and IBC to coexist in the same patient. In the present study, we describe a case of a 63-year-old Yemeni female patient diagnosed with locally advanced left breast cancer presented with pain at the left breast and axilla. A computed tomography (CT) scan for staging showed an incidental large hepatic mass, which was eventually discovered to be HAML. The patient underwent a modified radical mastectomy after completing her neoadjuvant treatment and later underwent parenchyma-sparing liver resection of that lesion; follow-up has continued till now. The diagnosis of HAML in the presence of IBC can pose challenges due to overlapping clinical and radiological features. Treatment decisions for patients with coexisting HAML and IBC require a multidisciplinary approach; surgical resection, embolization, targeted therapies, and systemic chemotherapy may be considered based on the extent of the disease and individual patient factors. Lastly, a brief review of the related literature was also carried out.

## Introduction

Hepatic angiomyolipoma (HAML) is a rarely seen type of mesenchymal tumor characterized by the presence of adipose tissues, smooth muscle cells, and blood vessels in different ratios [1].

Up to 2017, close to 600 cases of HAML were reported in the English literature [2]. Although HAML is more common in females, sex hormones do not appear to be involved in the pathophysiology or progression of the tumor [3]. The exact cause and pathogenesis of HAML are still unclear [3]. However, several risk factors and related conditions, such as tuberous sclerosis complex (TSC), have been associated with its occurrence [4].

HAML is usually asymptomatic and may be unintentionally found during routine medical exams or follow-up tests for other conditions [3]. Patients who experience HAML symptoms typically report abdominal discomfort, distension, and weight loss [2]. Because HAML has no clinical, laboratory, or radiographic characteristics, it is easily misdiagnosed as other forms of hepatic masses [5]. The vast majority of HAML cases are thought to be benign. However, a few cases of malignant behavior have been reported, such as growth, recurrence after surgical removal, metastasis, and invasive growth patterns in the hepatic tissue [6].

Inflammatory breast cancer (IBC) is an uncommon and extremely aggressive type of breast cancer, representing 2% to 4% of all breast cancer cases. Interestingly, liver lesions were observed in almost half of the individuals with metastatic cancer [7].

A limited number of cases have been described in the literature regarding the presence of HAML and IBC [8,9]. In Saudi Arabia, no previous study has been reported. Here, we report a case of a 63-year-old female who presented with IBC and was later diagnosed with HAML.

## Case presentation

A 63-year-old Yemeni female patient who has a history of diabetes mellitus and hypertension has been diagnosed with locally advanced left breast cancer. She presented with pain in the left breast and axilla and was admitted to our hospital, for treatment. The patient was alert, conscious, cooperative, airway patent, not hypoxic, and hemodynamically stable on physical examination. Breast examination was significant for ‏left breast at 11 o'clock position, lobulated mass, measuring about 5.8 cm x 2.8 cm, peau d'orange, nipple retraction, skin nodule, scar of previous biopsy, and left axillary lymph node enlargement. The right breast examination is unremarkable. The abdomen was lax and soft, with no tenderness. However, there was mild hepatomegaly, upper limb + lower limb power 5/5 bilateral felt pulses, no sign of deep vein thrombosis (DVT), and no lower limb oedema. Laboratory values, including serum bilirubin and transaminases (aspartate transaminase, alanine transaminase), blood urea, nitrogen, and serum creatinine, were all within the normal range, indicating normal liver and renal function serum tumor markers, cancer antigen 15-3 (CA15-3) was 33.7 U/mL (reference range: 0.0-31.3 U/mL).

A CT scan of the chest and abdomen was performed for staging purposes, and this showed left side breast with multi-focal heterogeneously dense breast lesions which are ill-defined in shape and irregular in margins, the largest was 2 cm x 3.1 cm x 3.3 cm in anteroposterior (AP), transverse, craniocaudal diameters respectively with mild decrease in size in comparison to the last study, associated with nipple retraction, skin thickening, surrounding free fluid and enlarged ipsilateral, and positive left axillary lymph nodes. The right breast also shows heterogenous ill-defined soft tissue density, the largest measuring 0.8 cm with no appreciated focal masses; the right axillary lymph nodes are still seen. 

**Figure 1:**
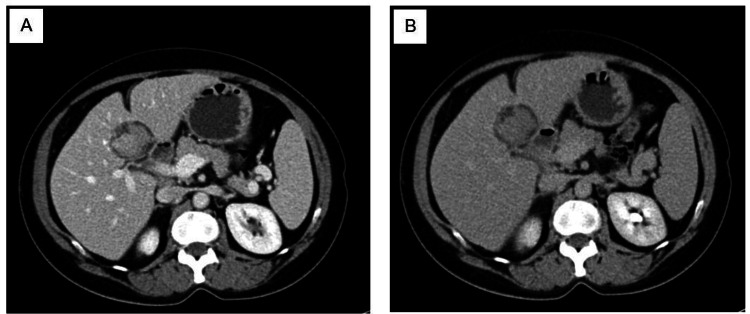
CT findings during arterial (A) and venous phase (B) showed a well-defined focal lesion measuring about 3.3 cm x 3.2 cm x 3.9 cm.

Furthermore, a suspicious solitary large hepatic focal lesion was seen as inseparable from the undersurface of the liver close to the interlobar fissure. Moreover, it appeared lobulated homogeneous centrally with surrounding hypodensity likely representing the target appearance. The lesion measured about 3.3 cm x 3.2 cm x 3.9 cm in AP, transverse, and craniocaudal dimensions, respectively, causing a mass effect (Figure 1).

**Figure 2:**
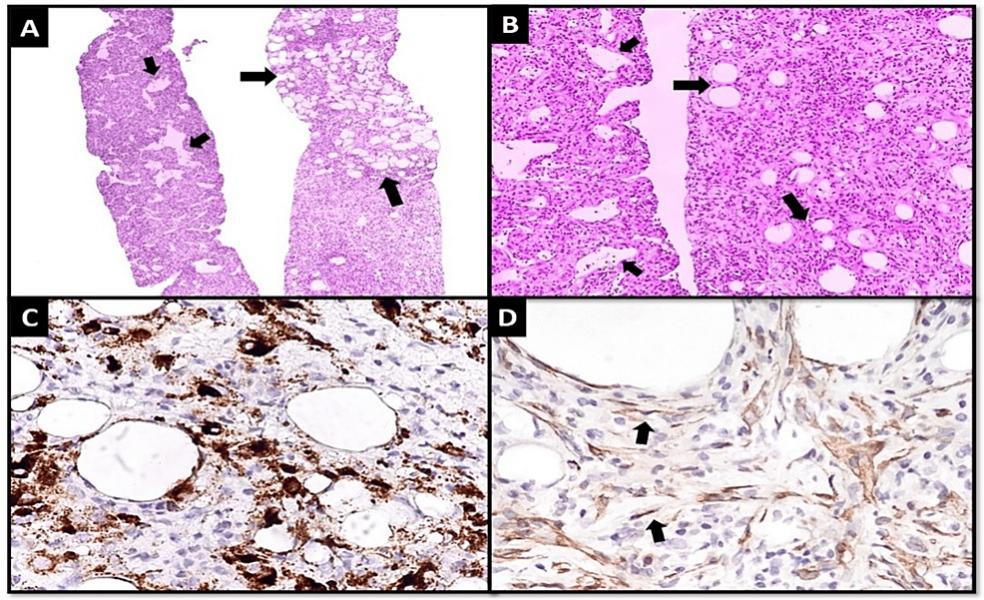
Tru-cut biopsy; (A) low power and (B) medium power views of the tumor, predominantly composed of sheets and fascicles of spindle cells with intervening collections of adipocytes with clear cytoplasm (larger arrows) and intervening thin-walled blood vessels (smaller arrows), (C) focal expression for HMB45 IHC stain is seen in epithelioid shaped tumor cells, and (D) focal expression for SMA IHC stain is seen in spindle-shaped tumor cells. SMA: Smooth muscle actin, IHC: Immunohistochemistry.

In addition, it was mentioned in the report as metastatic, but after the radiology review, there was fat content, and it looked mostly like angiomyolipoma rather than metastasis. A second opinion from the radiology side was taken, so we considered it a nonmetastatic benign lesion for follow-up. A core biopsy of the hepatic lesion was done to rule out metastatic breast lesions, so the diagnosis of angiomyolipoma was made (Figure 2).

**Figure 3:**
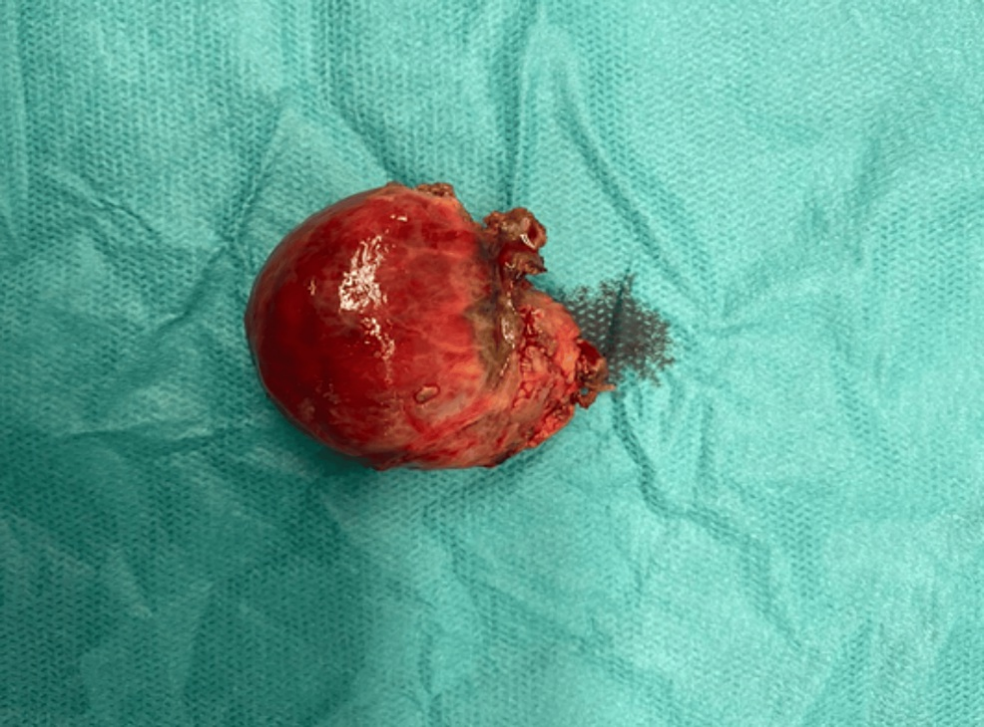
Angiomyolipoma, surgical specimen.

**Figure 4:**
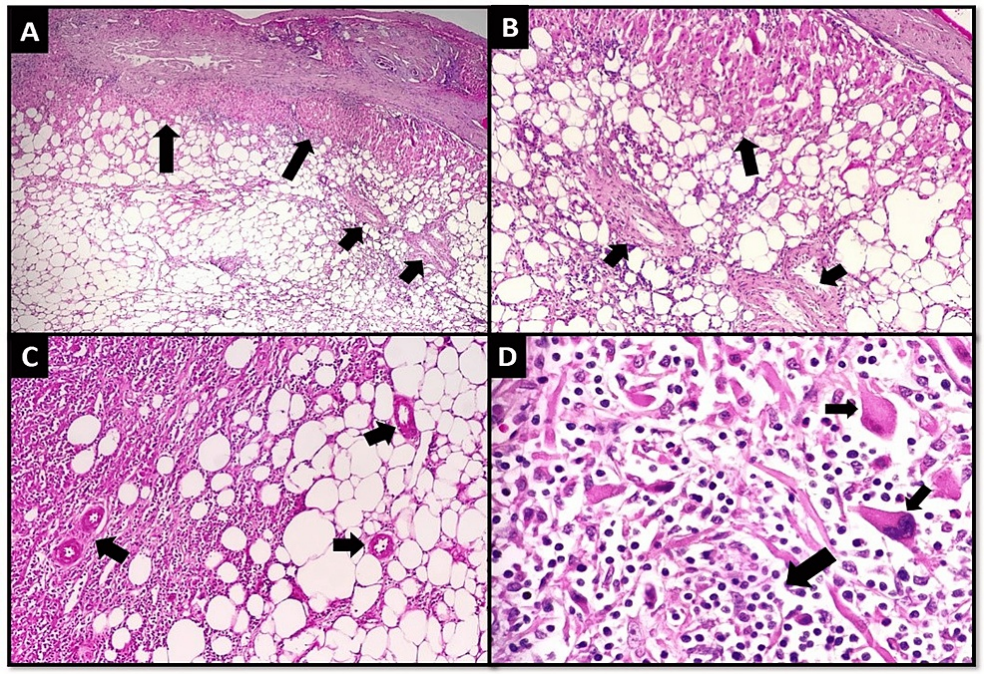
Excision specimen; (A) low power and (B) medium power views of the tumor invading the residual native liver tissue (larger arrows), with the tumor predominantly showing adipocytes with admixed spindle to epithelioid cells and thick-walled blood vessels (smaller arrows), (C) tumor cells showing spindle to epithelioid cells (left), adipocytes (right) and interspersed blood vessels (arrows), and (D) high power view showing epithelioid tumor cells (smaller arrows) and prominent lymphocytic infiltrate (larger arrow).

Histopathology of left breast Tru-cut biopsy revealed invasive carcinoma of no special type (ductal) histologic grade II (Nottingham histologic score: 6/9; tubule formation: 2; nuclear pleomorphism: 2; mitotic count: 2; ductal carcinoma in situ: present, <5%, solid type, nuclear grade II. The patient underwent a modified radical mastectomy after completing her neoadjuvant treatment and later underwent parenchyma-sparing liver resection of that lesion (Figure 3) and was sent for histopathology (Figure 4). The patient was discharged in good condition.

## Discussion

In the current study, we report a case of IBC which was later diagnosed with HAML. The patient underwent hospitalization for comprehensive staging and therapeutic interventions after presenting with pain in the left breast and axilla. Both breast cancer and HAML were confirmed by histopathology, and the left breast biopsy showed invasive carcinoma. A liver lesion biopsy revealed angiomyolipoma. Following the completion of neoadjuvant therapy, the patient underwent a modified radical mastectomy, followed by resection of the hepatic lesion.

In the previous literature review, there is a case documented in 2017 of a 37-year-old woman with a history of breast cancer who received a diagnosis of HAML. The patient had reported a history of abdominal pain for three months, which was not found to be associated with food intake, bowel movements, or body positioning and did not radiate to any site. Additionally, the patient experienced an unexplained weight loss of 3 kg. Upon physical examination, she had a tender abdomen, and a scar resulting from a previous left radical mastectomy was noted [8].

Another case was described in 2019, involving a 33-year-old female with a history of invasive breast carcinoma who was admitted to the hospital for elevated alpha-fetoprotein level. The patient displayed no signs or symptoms of abdominal discomfort or distension. Physical examination revealed only a scar from a modified radical mastectomy in the left breast [9].

 In comparison with our case, our patient was a 63-year-old female known case of breast cancer who presented to our hospital with left breast pain and was admitted for staging and treatment. Physical examination was significant for left multiple breast masses, Peau d'orange, nipple retraction, skin nodule, and scar of the previous biopsy.

HAML is a benign tumor mainly affecting middle-aged females with a 1:5 male-to-female ratio. It is usually discovered incidentally during medical check-ups since most patients do not experience any symptoms [2]. Nonetheless, a large tumor may cause abdominal discomfort, distention, and abdominal mass during examination [10].

In the study conducted by Dong et al., a CT scan revealed the presence of a nodule measuring 7.7 cm x 7.2 cm in the left hepatic lobe [8]. In our case, a pan CT scan demonstrated multifocal heterogeneously dense breast lesions on the left side, which are ill-defined in shape and irregular in the margin. Also, the liver enlarged with suspicious solitary large hepatic focal lesions and the presence of fat content. The lesion was approximately 5 cm in AP, transverse, and craniocaudal dimensions. Due to its varied composition, particularly its frequent paucity of fat, HAML can be easily mistaken for other malignant tumors during imaging examinations [11]. Additionally, the diagnosis of HAML is more difficult when invasive cancer is present, like in our case, because it is difficult to distinguish between HAML and metastasis [12]. Histological examination remains the definitive method for diagnosing HAML, considering the challenges associated with imaging techniques [2]. Therefore, a biopsy of the hepatic lesion confirmed the diagnosis of angiomyolipoma over the metastatic tumor. Immunohistochemistry analysis showed positive for HMB45, Actin, and KIT (CD117). A breast biopsy was taken from the left breast, which revealed invasive ductal carcinoma. Our patient had a modified radical mastectomy and a parenchyma-sparing liver resection of the lesion after her neoadjuvant therapy was finished, and the follow-up continued. In the study by Dong et al., laparoscopic hepatectomy was done as a diagnostic and curative procedure, proving the HAML diagnosis [8]. In the study of Zhang et al., complete laparoscopic resection of the right hepatic lesion was performed and sent to pathology, which confirmed the diagnosis of HAML [9]. After the procedure, both patients were discharged from the hospital without any problems and had not complained of any symptoms [8,9].

The optimal treatment option for HAML is still controversial despite the description of several therapeutic strategies [3]. Surgical resection is the preferred method by many researchers over a non-surgical approach that involves regular follow-up [3]. A non-surgical approach is also advised, particularly for patients with small tumors, no symptoms, or those considered unfit surgical candidates [3]. This method often entails frequent imaging with contrast-enhanced magnetic resonance imaging and close follow-ups [3].

Moreover, it is necessary to have a multidisciplinary team that includes hepatologists, radiologists, and pathologists [13]. A liver biopsy is recommended for a proper assessment [13]. Despite the rarity of HAML and IBC co-occurrence, clinicians need to be aware of this association and consider HAML when diagnosing liver tumors in patients with IBC.

## Conclusions

The coexistence of hepatic angiomyolipoma (HAML) and inflammatory breast cancer (IBC) is extremely rare. However, in cases of breast cancer and incidental hepatic mass, HAML should be considered as one of the differential diagnoses. Diagnostic challenges and interdisciplinary collaboration are crucial in accurately diagnosing and managing these cases. Continued research efforts are essential to enhance our understanding of this association and guide clinical decision-making for patients with IBC and HAML.
